# Effect of Red Mud Addition on Electrical and Magnetic Properties of Hemp-Derived-Biochar-Containing Epoxy Composites

**DOI:** 10.3390/mi14020429

**Published:** 2023-02-11

**Authors:** Silvia Zecchi, Fabrizio Ruscillo, Giovanni Cristoforo, Mattia Bartoli, Griffin Loebsack, Kang Kang, Erik Piatti, Daniele Torsello, Gianluca Ghigo, Roberto Gerbaldo, Mauro Giorcelli, Franco Berruti, Alberto Tagliaferro

**Affiliations:** 1 Department of Applied Science and Technology, Politecnico di Torino, C.so Duca degli Abruzzi 24, 10129 Torino, Italy; 2 Center for Sustainable Future Technologies, Italian Institute of Technology, Via Livorno 60, 10144 Torino, Italy; 3 Consorzio Interuniversitario Nazionale per la Scienza e Tecnologia dei Materiali (INSTM), Via G. Giusti 9, 50121 Firenze, Italy; 4 Department of Chemical and Biochemical Engineering, Institute for Chemicals and Fuels from Alternative Resources (ICFAR), Western University, London, ON N6A 5B9, UK; 5 Istituto Nazionale di Fisica Nucleare, Sez. Torino, Via P. Giuria 1, 10125 Torino, Italy

**Keywords:** biochar, red mud, conductive composites, co-pyrolysis, microwave

## Abstract

Waste stream valorization is a difficult task where the economic and environmental issues must be balanced. The use of complex metal-rich waste such as red mud is challenging due to the wide variety of metal oxides present such as iron, aluminum, and titanium. The simple separation of each metal is not economically feasible, so alternative routes must be implemented. In this study, we investigated the use of red mud mixed with hemp waste to produce biochar with high conductivity and good magnetic properties induced by the reduction of the metal oxides present in the red mud through carbothermal processes occurring during the co-pyrolysis. The resulting biochar enriched with thermally-reduced red mud is used for the preparation of epoxy-based composites that are tested for electric and magnetic properties. The electric properties are investigated under DC (direct current) regime with or without pressure applied and under AC (alternating current) in a frequency range from 0.5 up to 16 GHz. The magnetic measurements show the effective tailoring of hemp-derived biochar with magnetic structures during the co-pyrolytic process.

## 1. Introduction

The production of conductive composite materials is one of the most cutting-edge fields of materials science [1]. During the last decades, the scientific community has been focused on the great challenge represented by the exploitation of graphene and related materials [2] without solving the issue related to both cost and large-scale production [3,4]. Other carbonaceous fillers such as carbon fibers have gathered similar interest but they remained confined to high-technological fields such as the aeronautic industry [5]. Nonetheless, the research of new carbon-based fillers able to reach the same performances as costly nanostructured ones remains a crucial research topic. Biochar (BC) represents a valid solution to fulfill the ambitious aim to produce a price-affordable, high-performance conductive carbon composite [6,7]. BC is the solid product of the pyrolytic conversion of biomass, representing a highly-tunable carbon source for material science applications [8]. Recently, BC has been successfully used to produce thermoset [9,10] and thermoplastic composites [11,12,13,14] with appreciable microwave (MW) absorption properties. As reported by Torsello et al. [15], the electrical properties of BC-containing composites are strongly correlated with the temperature used for BC production. Nevertheless, there are technical limitations to the maximum temperature that can be reached during a pyrolytic process [16,17]. These limitations represent a weakness for the performance improvement of BC-based composites used for high-frequency shielding that require a high conductivity, such as those reached for processing temperatures above 1000 °C [18] for matching high-tech nanostructured carbon materials [19,20,21,22,23,24,25,26].

The increment of electrical conductivity is not the only route to boost the MW shielding effectiveness, and the inclusion of magnetic metals [27] beneficially affects the final properties of the composite. The metallic species used for the MW shield are generally iron-based nanoparticles [28,29,30] or complex metal oxides [31,32,33] that are not easy to produce and remarkably costly. Nevertheless, the production of tailored BC by a simple co-pyrolytic process of biomass with iron-rich waste could be a solid approach to producing magnetic BC through carbothermal processes [34,35,36,37]. Among the various sources, red mud (RM) is a widely-available and iron-rich waste stream produced from the Bayer process of the aluminum refinery from bauxite [38] that is hard to dispose of or reuse [39,40]. The metal recovery from RM through the metallurgic process is quite expensive [41,42,43,44] and the use of neat RM will be preferred for the production of inorganics materials [45,46,47]. In this work, we studied the co-pyrolysis of RM with hemp crop wastes from the production of a magnetic conductive filler (RM-BC) for the preparation of epoxy composites. Hemp was selected due to its large availability and the high aspect ratio of the tiny hemp fibers that are not used in the hemp-based value chain. Several mixing feedstocks were co-pyrolyzed in a two-stage process reaching a final temperature of 900 °C. A comprehensive electrical and magnetic characterization was carried out to investigate the properties of the neat BC filler.

## 2. Materials and Methods

### 2.1. Materials

Hemp and Red Mud were provided by The Institute for Chemicals and Fuels from Alternative Resources (ICFAR, London, ON, Canada). Two-component Bisphenol A (BFA) diglycidyl resin was purchased from CORES (Cores epoxy resin, Lendinara, Italy).

### 2.2. Methods

#### 2.2.1. RM-BC Production

Co-pyrolysis of hemp and red mud was run by mixing the feedstock using a red mud wt.% of 100 wt.%, 50 wt.%, 20 wt.%, 10 wt.%, 5 wt.% and 0 wt.% respectively. RM-BC was produced by using a two-stage route. In the first step, 1 kg of feedstock was pyrolyzed in a mechanical fluidizing bed reactor run at 600 °C for 1 h using a heating rate of 15 °C/min with a set temperature of 600 °C. The inert gas flow (nitrogen) was set at 1 L/min while the shaft spin was set at 20 RPM. Every run the reactor is loaded with 150 g.

During the second step, 150 g of BC produced during pyrolysis were annealed in a static furnace at 900 °C using a heating rate of 5 °C/min. Once the annealing temperature was reached, the annealing process lasted 2 h. The annealing process was run in a carbon dioxide (flow rate: 1 L/min) nitrogen (flow rate: 2 L/min) mixed atmosphere leaving the system in the same condition for the cooling down.

#### 2.2.2. RM-BC Containing Epoxy Composites Production

RM-BC-based composites containing 900 °C-annealed materials were prepared according to the report of Bartoli et al. [48]. RM-BC samples were mechanically pulverized and subsequently dispersed into the epoxy monomer using a tip ultrasonicator apparatus (Sonics Vibra-cell, Neton, MA, USA) for 15 min. To avoid an excessive temperature rise, ultrasounds were pulsed with cycles of 20 s alternating with pauses of 10 s to allow better heat diffusion. After the addition of the curing agent, the mixture was further ultrasonicated for 2 min and left in the molds for 16 h at room temperature. A final thermal curing was performed using a ventilated oven (I.S.C.O. Srl “The scientific manufacturer”, San Donà di Piave, Italy) at 70 °C for 6 h. The concentration of RM-BC in the composites was 30 wt.% for all the materials.

#### 2.2.3. RM-BC and RM-BC Containing Composites Characterization

Ultimate analyses of RM-BC were run by using a Thermo FlashEA^®^ 1112 unit (Thermofisher, Waltham, MA, USA) adding vanadium pentoxide (Sigma Aldrich, St. Louis, MO, USA) to the samples to evaluate the amount of sulfur.

Proximate analyses were run accordingly with the ASTM methodology (ASTM D1762-84).

The surface area and pore size analyses of RM-BC were run by using a Quantachrome NOVA 2000e (Microtrac, Japan) and the BET model for the data interpretation.

Raman spectra of RM-BC samples were collected using a Renishaw inVia (H43662 model, Gloucestershire, UK) equipped with a green laser line (514 nm) with a 50× objective. Raman spectra were recorded in the range from 250 cm^−1^ to 3500 cm^−1^. The decomposition of Raman spectra was focused on the range 1000–2000 cm^−1^ and performed with homemade software developed using Matlab^®^ (version R2020a, The Mathworks, Inc., Natick, MA, USA) according to the procedure proposed by Tagliaferro et al. [49].

All RM-BC samples were investigated from the morphological point of view using a field-emission scanning electron microscope (FE-SEM, Zeiss SupraTM40, Oberkochen, Germany). The microscope was equipped with an energy-dispersive X-ray detector (EDX, Oxford Inca Energy 450, Oberkochen, Germany) that was used to explore the RM-BC composition of biochars.

The DC electrical conductivity of the RM-BC composites was determined by measuring the electrical resistance *R* of samples of regular parallelepiped shape (length *l*, width *w,* and thickness *t*) at room temperature. Values of *R* smaller than 10 MΩ were measured in the four-wire van der Pauw configuration using a 34410A digital multimeter (Keysight Technologies, Santa Rosa, CA, USA) by drop-casting small PELCO^®^ silver paste (TedPella, Redding, CA, USA) contacts on the corners of the samples; the conductivity was then determined as $\sigma =1/R_{s}t$, where $R_{s}$ is the sheet resistance of the samples obtained by solving the van der Pauw equation from the values of *R* measured in the horizontal and vertical configurations [50]. Values of *R* larger than 10 MΩ were measured in the two-wire configuration using a 6517B electrometer (Keithley Instruments, Solon, OH, USA) by completely covering the two facets at distance *l* of the samples with PELCO^®^ silver paste contacts; the conductivity was then determined as $\sigma =R^{-1}t^{-1}w^{-1}l$.

The electrical resistance of composites was also measured under increasing pressures (up to 750 bar) applied by a hydraulic press (Specac Atlas Manual Hydraulic Press 15T, Orpington, UK) according to Giorcelli et al. [10]. Electrically insulating sheets were placed between the conductive cylinders and the load surfaces to ensure that the electrical signal went through the sample. The resistance of the carbon fillers was measured using an Agilent 34401A multimeter (Keysight Technologies, Santa Rosa, CA, USA).

The complex permittivity of the samples was measured in the GHz range by means of a cylindrical coaxial cell (EpsiMu toolkit [51]), containing the sample as a dielectric spacer between inner and outer conductors, whose diameters are 0.6 cm and 1.3 cm, respectively. Two conical parts link the cell to standard connectors, allowing it to keep the characteristic impedance to 50 Ω, thus avoiding mismatch and energy loss. The cell is connected to a Rohde Schwarz ZVK Vector Network Analyzer (Colorado Springs, CO, USA), suitably calibrated, and measurements are analyzed with a two-port transmission line technique. The electromagnetic properties of the sample are determined by de-embedding and the Nicolson-Ross-Weir transmission/reflection algorithm [52,53].

Magnetic properties were investigated with a DC magnetometer/AC susceptometer (Lakeshore 7225, Westerville, OH, USA) equipped with an electromagnet at room temperature in quasi-static conditions. In particular, magnetic hysteresis cycle measurements were performed on the composite samples up to 30 kA/m to estimate the main magnetic parameters of the materials (i.e., magnetic susceptibility). The mass magnetic susceptibility χ_ρ_ is computed as the slope of the low-field first magnetization branch of the hysteresis loop. The composite samples' weight was measured and the filler wt.% is known from the preparation, allowing us to obtain the magnetic characterization of the filler alone, since the signal from the polymeric matrix is negligible [15].

## 3. Results

### 3.1. Characterization of RM-BC Materials

#### 3.1.1. Ultimate, Proximate, and Surface Analysis

**Figure 1:**
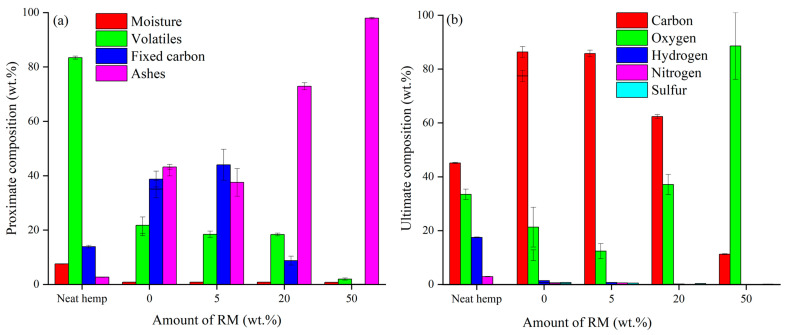
(**a**) Proximate and (**b**) ultimate analysis of RM-BC materials.

A preliminary evaluation of RM-BC materials produced was run through proximate and ultimate analysis as shown in Figure 1.

**Figure 2:**
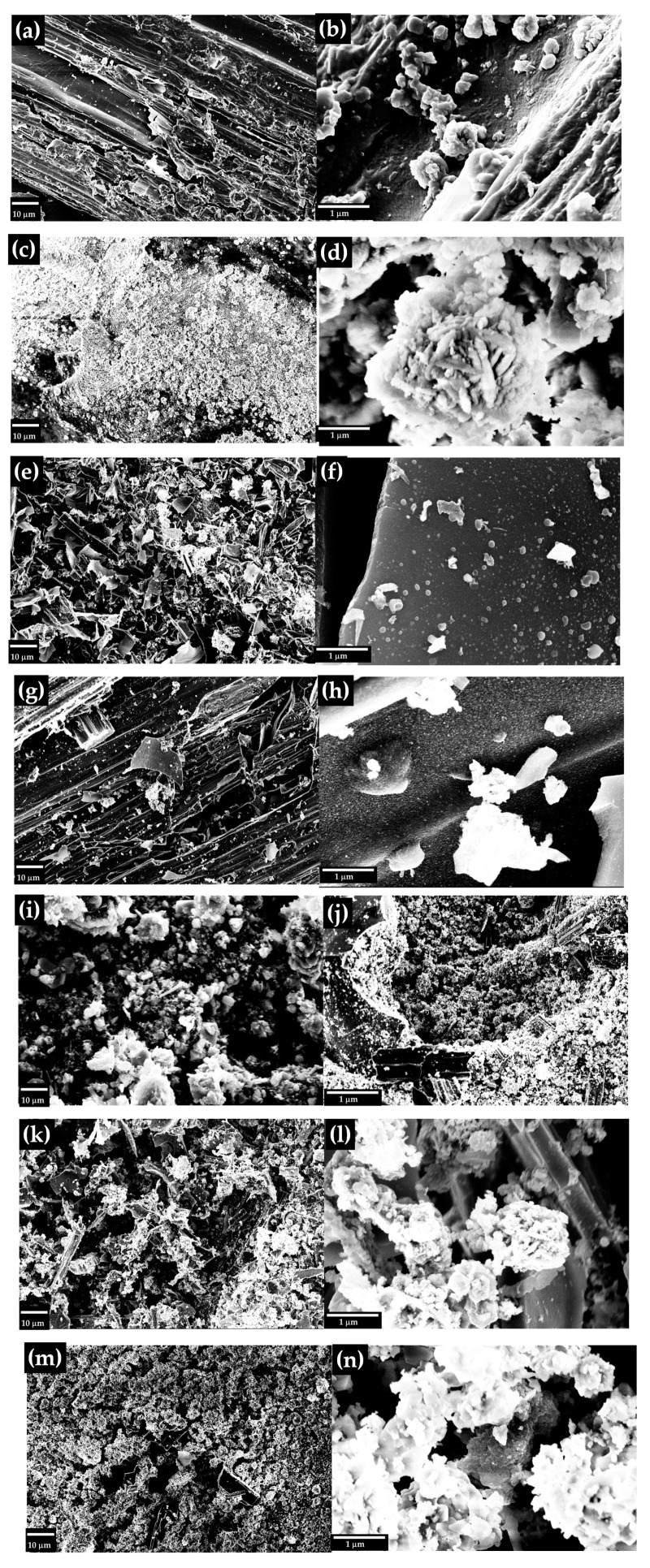
FE-SEM images of (**a**,**b**) neat hemp, (**c**,**d**) neat RM and RM-BC materials with (**e**,**f**) 0 wt., (**g**,**h**) 5 wt.%, (**i**,**j**) 10 wt.%, (**k**,**l**) 20 wt.% and (**m**,**n**) 50 wt.% of RM respectively.

As reported in Figure 1a, neat hemp showed a high number of volatiles up to 85.4 ± 0.5 wt.% and a low ash content up to 2.7 ± 0.1 wt.%, while the BC produced without the addition of RM showed an amount of volatiles up to 19.8 ± 1.9 wt.%. By increasing the RM amount up to 20 wt.%, the volatiles did not significantly change while using 50 wt.% of RM they decreased to 2.9 ± 0.4 wt.%. Fixed carbon decreased by increasing the RM amount while the ash content displayed an opposite trend reaching 97.8 ± 1.0 wt.% using 50 wt.% of RM. This agreed with the oxygen content observed in the ultimate analysis reported in Figure 2b. Oxygen content showed a drastic increment moving from neat BC to the RM-BC produced by adding 50 wt.% of RM, where a value of up to 88.6 ± 12.4 wt.% was reached. These data suggest that the increment of RM during the co-pyrolytic process led to the accumulation of inorganic into the RM-BC materials. The great amount of oxygen detected was reasonably due to the massive presence of oxides.

Surface analysis textures of RM-BC samples were also investigated, and the main outputs are summarized in Table 1.

As reported in Table 1, the surface properties of RM-BC materials were deeply related to the amount of RM added during the co-pyrolysis. RM-BC samples produced by using a 0 and 5 wt.% of RM showed surface area up to 250 and 237 m^2^/g respectively with pore volume and average pore radius totally comparable. By increasing the RM amount, the surface area and pore volume decreased down to 20 m^2^/g and 0.02 cm^3^/g when 50 wt.% of RM was used while the average pore radius increased up to 1.7 nm. These data suggest a collapsing of the porous network due to the advanced cracking reactions promoted by the presence of RM as reported by Lim et al. [54]. Furthermore, the increment of RM drastically increased the ash of the RM-BC materials suggesting the degradation of original hemp structures.

#### 3.1.2. Structural Analysis

Neat hemp, RM, and RM-BC morphologies were investigated by using FE-SEM as shown in Figure 2 while their elemental composition was evaluated also by using EDX as reported in Table 2.

As shown in Figure 2a,b, the neat hemp was composed of micrometric fibers with a channeled surface while RM (Figure 2c,d) was composed of lamellae of inorganic species with a thickness of around 150 nm. The RM-BC produced without adding RM shows a typical BC structure (Figure 2e,f) with the loss of the original morphology of neat hemp. By adding RM, the formation of nanoparticles on the surface of BC was observed (Figure 2h). These particles produced by carbothermal reduction of the oxides present in the RM become bigger and more numerous by increasing the amount of RM used, as shown in Figure 2j (RM 10 wt.%) and Figure 2l (RM 20 wt.%). As shown in Figure 2m,n, the addition of 50 wt.% of RM induced a morphology quite close to the one observed for the neat RM. This was reasonably due to the consumption of the organic matrix by the reduction of metal species, in agreement with ash and oxygen content amounts observed in the proximate and ultimate analysis. This process takes place at a temperature higher than 800 °C for Fe(III) [55] and partially converts the carbon matrix into CO and CO_2_ reducing the metal to zero valences. Accordingly, the removed carbon creates channels and pores inducing disruption of the original morphology of the hemp matrix.

Further insight into the RM-BC composition was provided by the EDX elemental analysis reported in Table 2.

Neat RM was mainly composed of aluminum and iron that reached 12.1 and 26.3 wt.% respectively. RM-BC materials showed an increasing amount of inorganic species and a decrement in carbon amount with the increment of RM, in agreement with the ultimate analysis. The mismatch between these two analytic approaches was mainly due to the volume investigated which was limited to a few micrometers in the case of EDX analysis [56] while the ultimate analysis was carried out on the bulk.

**Figure 3:**
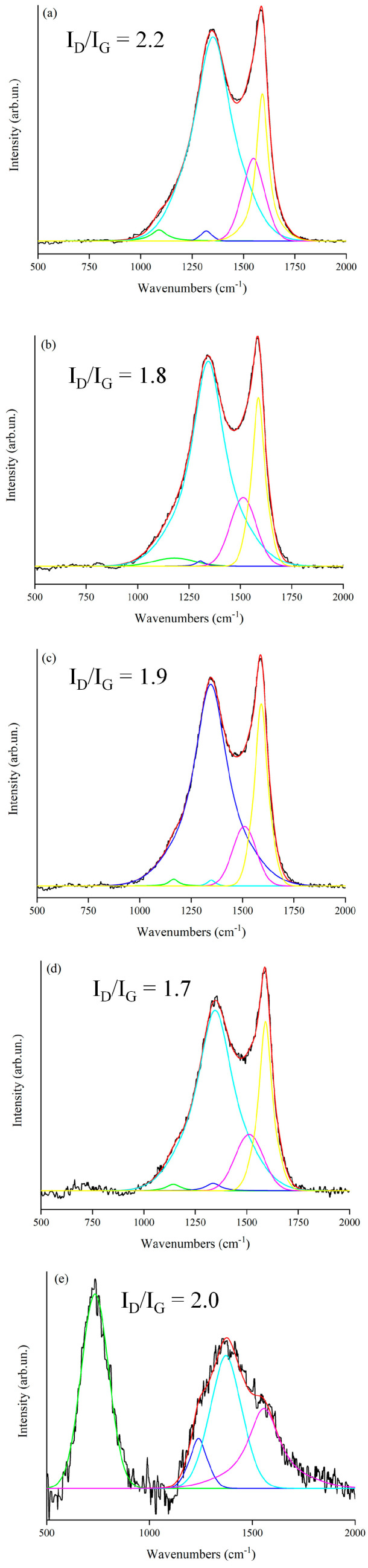
Raman spectra (black lines) in the range from 500–2000 cm^−1^ of RM-BC materials containing (**a**) 0 wt., (**b**) 5 wt.%, (**c**) 10 wt.%, (**d**) 20 wt.% and (**e**) 50 wt.%f RM respectively. Fitted signals (red lines) were obtained by using Gaussian-Lorentzian (GauL) or line-shaped components (colored lines), according to Tagliaferro et al. [49].

The degree of graphitization of the carbon structures of RM-BC was evaluated by using Raman spectroscopy as shown in Figure 3.

The Raman spectra of RM-BC materials were quite similar for RM amounts ranging from 0 up to 20 wt.% with I_D_/I_G_ values that are ranging from 1.7 up to 2.2. The RM-BC sample prepared by using 50 wt.% of RM showed a very different profile compared to common carbonaceous spectra [57]. This was reasonably due to the massive presence of inorganics as proved by the very intense and broad band centered at 789 cm^−1^ due to MO_x_ species (iron oxides, aluminum oxides). The formation of oxides is quite common after carbothermal reduction and it is due to the passivation of an external layer of metal structures as reported by Tamborrino et al. [34] for iron-tailored BC materials.

### 3.2. Characterization of RM-BC Composites

#### 3.2.1. DC Electrical Characterization

**Figure 4:**
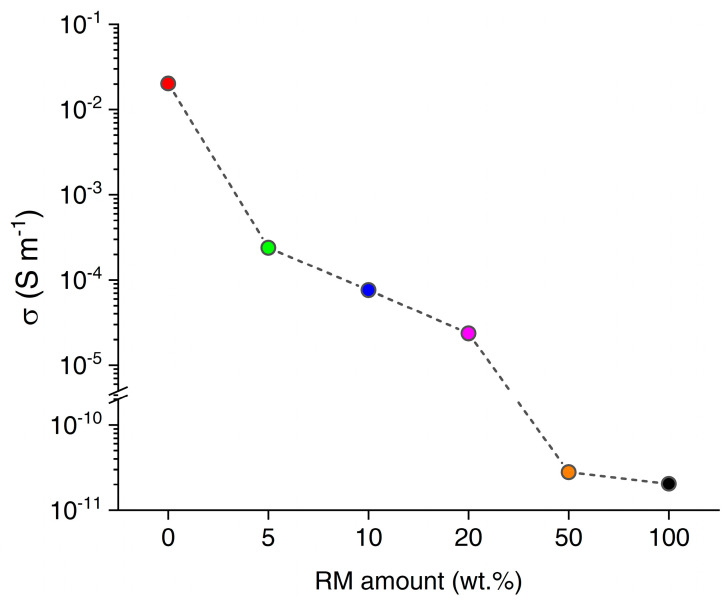
Electrical DC conductivity of RM-loaded epoxy composites in ambient conditions, as a function of the amount of RM used for the production of each filler (red 0 wt.%, green 5 wt.%, blue 10 wt.%, magenta 20 wt.%, orange 50 wt.%, black 100 wt.%).

The DC electrical conductivity of RM-BC composites was investigated in ambient conditions for increasing the RM amount in the filler, as shown in Figure 4. The highest conductivity of 20 mS/m was observed in the composite without RM. At the increase of the RM amount, the conductivity rapidly dropped to 240 μS/m (5 wt%), 76 μS/m (10 wt%), and 24 μS/m (20 wt%). Further increasing the RM amount led to a drastic reduction of the conductivity by 6 orders of magnitude to 28 pS/m (50 wt%), likely due to the reduced BC content leading to the disappearance of percolation through conductive carbon particles. Increasing the RM amount even further did not affect the conductivity appreciably (20 pS/m at 100 wt%).

**Figure 5:**
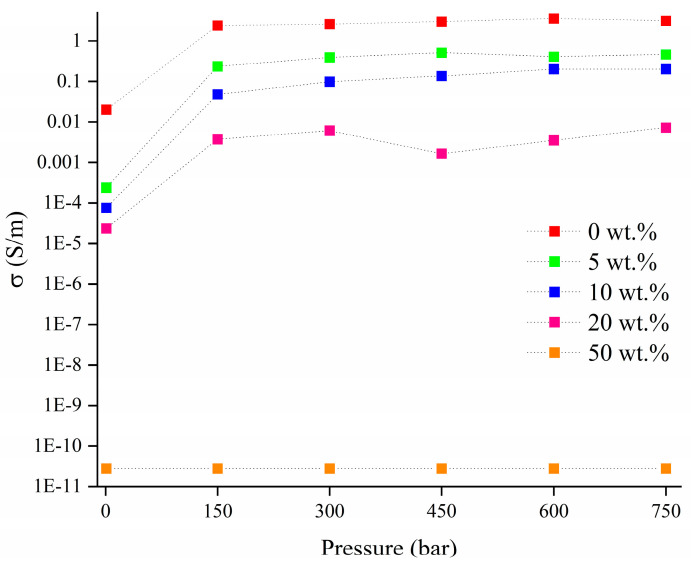
Conductivity measurement in a pressure range from 1 bar up to 750 bar of 30 wt.% RM-loaded epoxy composites. In the legend are reported the amounts of RM used for the production of each filler.

The electrical conductivity of RM-BC composites was also evaluated by applying pressure from 1 up to 750 bar as shown in Figure 5.

Composites produced by adding RM-BC obtained without RM showed the highest conductivity that reached 3.2 S/m at 750 bar, while by increasing the amount of RM the conductivity decreased reaching values of 0.4 and 0.2 S/m by using RM-BC produced by adding 5 and 10 wt.% of RM. Further increments of RM led to a further decrement of conductivity down to 0.007 S/m for RM-BS produced using 20 wt.% of RM. The samples containing RM-BC produced by adding 50 wt.% of RM and neat RM were too poor in conductivity to be measured by this setup.

#### 3.2.2. Magnetic Characterization

**Figure 6:**
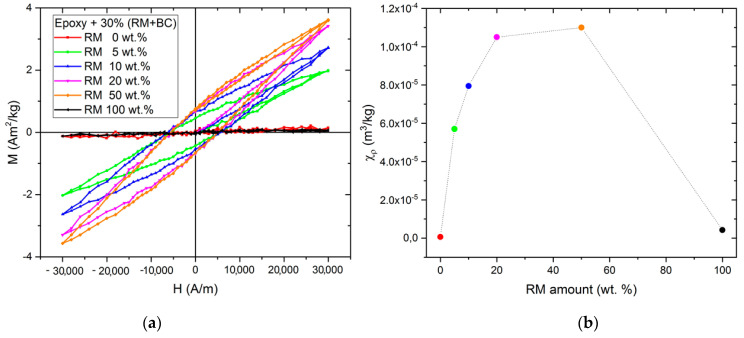
(**a**) Magnetization curves (hysteresis cycles) of the composite samples up to 30 kA/m and (**b**) χ_ρ_ vs red mud content in the filler of the composite samples.

The hysteresis cycles up to 30 kA/m for all the samples are collectively shown in Figure 6, together with the behavior of the magnetic susceptibility of the samples as a function of the biochar content in the filler.

Qualitative and quantitative differences can be noted between the composites with Red Mud or biochar alone (which are paramagnetic) and the composites in which the RM has been reduced in the synthesis by the carbon in the biochar. The latter samples show a typical ferromagnetic behavior with a clear hysteresis loop. As clearly visible from Figure 6b, the intensity of the magnetic features is strongly dependent on both the red mud and biochar content in the sample, yielding a non-monotonic behavior of the χ_ρ_ dependence on the filler composition inferior to the paramagnetic iron nanoparticles [58], that supports the consideration that the magnetic particles are obtained from the reduction of the oxides in the red mud.

#### 3.2.3. High-Frequency Electrical Characterization

**Figure 7:**
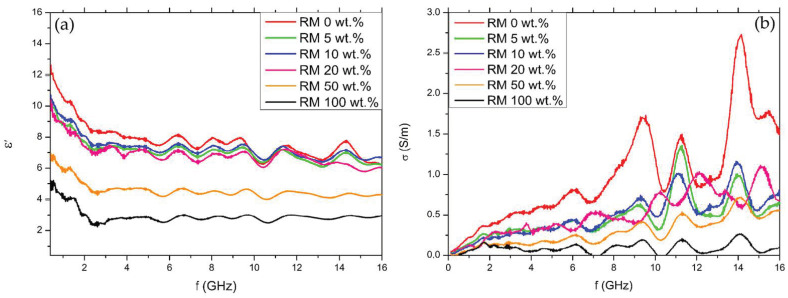
Real part of the complex permittivity (**a**) and conductivity (**b**) as a function of frequency.

The real part of the complex permittivity (ε’) and the conductivity (σ) are shown in Figure 7 as a function of frequency (f) in the measured range.

The trend of the curves of real permittivity is the same for each composition: for increasing frequency, there is an initial decrease of ε’, followed by a plateau starting from about 2.5 GHz. Similar fluctuations are observed in all curves and are due to the measurement setup, whereas the overall trend (i.e., the decrease and plateau) is due to the nature of the samples. Considering the conductivity, the curves exhibit a monotonically-increasing trend.

In Figure 7, it can be seen that, by increasing the ratio of red mud to biochar in the filler, the values of permittivity and conductivity tend to decrease as observed by Torsello et al. [15]. Nevertheless, an increase of these properties with the increase of the biochar content (which one would expect) can be seen in the samples with very different concentrations (0, 50, and 100 wt.%), whereas for more similar concentrations the curves tend to fall closer together. This can be related to the low sensitivity of the measurement: for samples with a similar amount of RM (5, 10, 20 wt.%) the variation between electromagnetic properties falls within experimental error.

## 4. Conclusions

In this study, we proved that the modification of the ratio between RM and hemp allowed us to enhance the electrical or magnetic properties of the material employed as a filler in composite samples. The increment of the RM-to-hemp ratio led to a significant reduction of conductivity of the resulting BC under both DC and AC regimes while it increased the magnetic signal of the composites. This was due to the insulating behavior of inorganic particles tailoring the BC particles. Accordingly, the addition of the same amount of filler led to a decrement in the conductive filler fraction. This was particularly evident for the addition of RM up to 10 wt.%. Nevertheless, the simultaneous good conductive and magnetic properties of BC-containing composites could represent a solid solution to produce microwave-shielding materials and magnetic-responsive composites. These high-value applications support the inclusion of RM into the thermochemical conversion of hemp leading the way for the virtuous use of complex waste streams.

## Figures and Tables

**Table 1:** Surface analysis outputs of RM-BC materials.

Amount of RM (wt.%)	Surface Area (m^2^/g)	Total Pore Volume (cm^3^/g)	Average Pore Radius (nm)
0	250	0.15	1.2
5	237	0.16	1.3
10	167	0.12	1.4
20	135	0.08	1.5
50	20	0.02	1.7

**Table 2:** EDX analysis of neat RM and RM-BC materials with 0 wt., 5 wt.%, 10 wt.%, 20 wt.% and 50 wt.% of RM respectively.

Element (wt.%)	RM Amount (wt.%)					
	*RM*	*0*	*5*	*10*	*20*	*50*
C	0.0	71.3	75.8	46.9	23.7	7.1
O	42.9	7.6	7.8	22.5	32.0	35.3
Na	6.1	0.0	0.0	2.5	3.6	4.2
K	0.3	0.0	9.0	2.8	3.7	0.9
Mg	0.0	3.3	0.5	0.7	0.6	0.0
Ca	0.6	5.2	2.5	5.2	2.5	4.8
Al	12.1	0.0	0.5	6.1	10.1	15.1
Si	6.8	0.0	0.7	2.8	5.1	6.1
P	1.5	0.0	0.0	0.0	0.0	0.0
S	0.0	1.0	0.0	0.4	0.0	0.0
Ti	3.5	0.0	3.4	1.9	2.4	4.2
Fe	26.3	0.0	0.0	8.1	16.3	22.4

## Data Availability

The data presented in this study are available on request from the corresponding authors.
